# Mine pressure behavior law of isolated island working face under extremely close goaf in shallow coal seam

**DOI:** 10.1038/s41598-023-47907-x

**Published:** 2023-11-23

**Authors:** Tianwei Lan, Yonghao Liu, Yongnian Yuan, Hongliang Liu, Hongguang Liu, Shunfeng Zhang, Shunxiang Wang

**Affiliations:** 1 College of Mining, Liaoning Technical University, Fuxin, China; 2 Ordos Institute of Liaoning Technical University, Ordos, China; 3 Inner Mongolia Limin Coal Co., Ltd., Wuhai Energy Group, CHN Energy, Ordos, China; 4 Inner Mongolia Baiyinhua Energy Co., Ltd., Inner Mongolia, Xilinguole China; 5 Shenhua Geological Exploration Co., Ltd., Beijing, China

**Keywords:** Solid Earth sciences, Geodynamics, Geology

## Abstract

In order to study the mining pressure characteristics of the shallow buried coal seam with the same silo working face under the very close mining void zone and the overlying coal rock body, the theoretical analysis is used to determine whether the open-cutting eye bearing layer belongs to the mining under the very close mining void zone or not, based on the numerical simulation of FLAC3D and on-site measurement of the working resistance at the end of the cycle of the working face's hydraulic bracket, It is proposed to divide the mining stress of the working face based on the advancing length of the working face, that is, the high-pressure zone, the transition zone and the low-pressure zone. The results of the study show that: FLAC^3D^ software was used to analyze the stress intensity of the “C” island working face when it was mined back to 50 m, 100 m, 150 m, and 180 m (one time “square”), and the simulation results were imported into the Origin software, which was used to analyze the stress intensity of the working face. The simulation results were imported into Origin software, and the influence range of mining stress was divided into four areas: high-stress area, stress transition area, low-stress area, and "square" stress concentration area. According to the on-site measurement of the working resistance at the end of hydraulic support cycle, the initial pressure step of the working face under the overlying coal rock body is 48.9–55.7 m, with the peak value of 38 MPa, the cycle pressure step is 9.0–23.3 m, with the peak value of 36 MPa, and the dynamic load factor of the working face is 1.14–1.16; relative to the overlying coal rock body, the average decrease of the cycle pressure step is nearly 10% and the average increase of dynamic load factor is 1.14–1.16; compared with that under the overlying coal rock body, the average decrease of the cycle pressure step is nearly 10% and the average increase of dynamic load factor is 1.14–1.16. Compared with the overlying coal rock body, the average decrease of the cycle pressure step under the overlying mining zone is nearly 10%, the average increase of the dynamic load factor is 20%, and there is no obvious regularity and periodicity in the direction of strike, and the working face is divided into three parts along the direction of strike: high-pressure zone, transition zone, and low-pressure zone. Therefore, in the process of mining under the overlying coal rock body, we should strengthen the roadway peripheral rock support and roof and floor management, which is of guiding significance to the mining of similar working faces and mine safety production.

## Introduction

The practice has proved that the shallowly buried coal seam mining pressure is not necessarily small, longwall face generally appears to have the phenomenon of step down, mining pressure manifestation is intense, shallowly buried coal seam working face roof breaking movement has special characteristics^1–3^, for the very close distance coal seam mining is mainly used in the stratified mining, coal seam mining, and comprehensive mining top coal mining method^4^, therefore, the research on the working face of the law of the manifestation of the mining pressure has entered a new stage.

Aiming at different geological conditions, different mining areas, different coal seams under the coal seam mining technology program selection, mine pressure manifestation, and surrounding rock control, related scholars have carried out a lot of research work. Zhang Zhi^5^ and other complementary Lianta 11401 comprehensive mining face as the object, research shallow buried coal seam super-long working face mining pressure law, the study shows that the working face to pressure distribution is uneven, asynchronous, the working face to pressure during the roadway over the destruction of the degree of small, the roof collapse of the mining area is more stable. Fu et al.^6^ take Shendong mine as the research background, use theoretical analysis and field measurement methods to study the characteristics of the working face under the very close distance mining hollow area, and the research results show that the “masonry beam” structure formed under the overlying rock layer on the working face is more likely to be destabilized, and the incoming pressures are frequent and the incoming pressures are of high intensity. Fan Dechao et al.^7^ studied the evolution of surrounding rock stresses during the mining period based on a large-scale geomechanical model test system, and analyzed the distribution characteristics of surrounding rock stresses under different widths of coal pillars. Chen et al.^8^ used FLAC^3D^ numerical simulation to systematically analyze the vertical stress and horizontal displacement of right-angle trapezoidal roadway, revealing the distribution law of surrounding rock stress and damage characteristics of the right-angle trapezoidal roadway, and the results showed that the larger the surrounding rock stress the larger the inclination angle of the roadway, and the two gangs of the roadway, the roof plate and the sharp corners showed obvious asymmetry of the stress concentration phenomenon. Wang et al.^9^ used a numerical simulation method to analyze the top and bottom plate stress law before and after mining back to the working face of the isolated island, the study found that the roadway distance from the bottom plate distance increases, the degree of roadway stress concentration in the surrounding rock shows a decreasing trend, the top and bottom plate stress is released after the working face is stepped back and provides the theoretical basis for the subsequent support of the surrounding rock of the roadway. Other related scholars have carried out in-depth research on the overtopping coal pillar instability^10^, surrounding rock stress^11^, mining technology^12,13^, disaster prevention and control^14^, etc., but the same working face is in the very close mining conditions, there are two kinds of endowment conditions at the same time under the overlying coal and rock entities and overlying air-sea areas, and the working face is in the However, there are fewer studies on the characteristics of mineral pressure manifestation in the area of “C” island working face.

The author combined with Baiyinhua mine shallow buried coal seam very close to the mining area under the island working face mining specific conditions, using theoretical analysis, numerical simulation, and on-site measurement methods, compared and analyzed the mining face in the overlying coal rock entity under the overburden and under the overburden of the mining airspace under the law of the emergence of the mine pressure, this study can be similar conditions for the mining of the island working face to provide guidance and reference.

## Engineering background

Inner Mongolia Baiyinhua mine 1202-2 synthesized working face is located in the first level of the second mining area, mining and releasing ratio 1:3, working face elevation + 654.2 to  + 723.4 m. Depth of burial 260–308 m, average depth of burial 284 m, working face strike length 2347 m, inclined length 180 m. The average thickness of the coal bed is 18 m, and the angle of inclination of the coal bed is 5–8°. From the opening eye 0–107 m is located under the solid structure of the overlying coal rock, the rest is located under the overlying air space. The average thickness of the coal seam is 18 m, and the dip angle of the coal seam is 5–8°. The working face is located under the solid structure of the overlying coal and rock from 0  to  107 m of the cutting eye, and the rest of the working face is located under the overlying air-mining zone.

The 1202 working face of the overlying mining area was finished mining back in May 2016, the average thickness of the coal seam is 18 m, the mining and releasing ratio is 1:3, the top plate lithology is dominated by mudstone, which is basically stable, and the overlying rock strata present a single structure, the 1202 working face mining leads to the periodic breakage of the key layer, and there is a 1 m mezzanine layer between the two workfaces, and the two workfaces belong to the extremely close distance mining workface.

**Figure 1:**
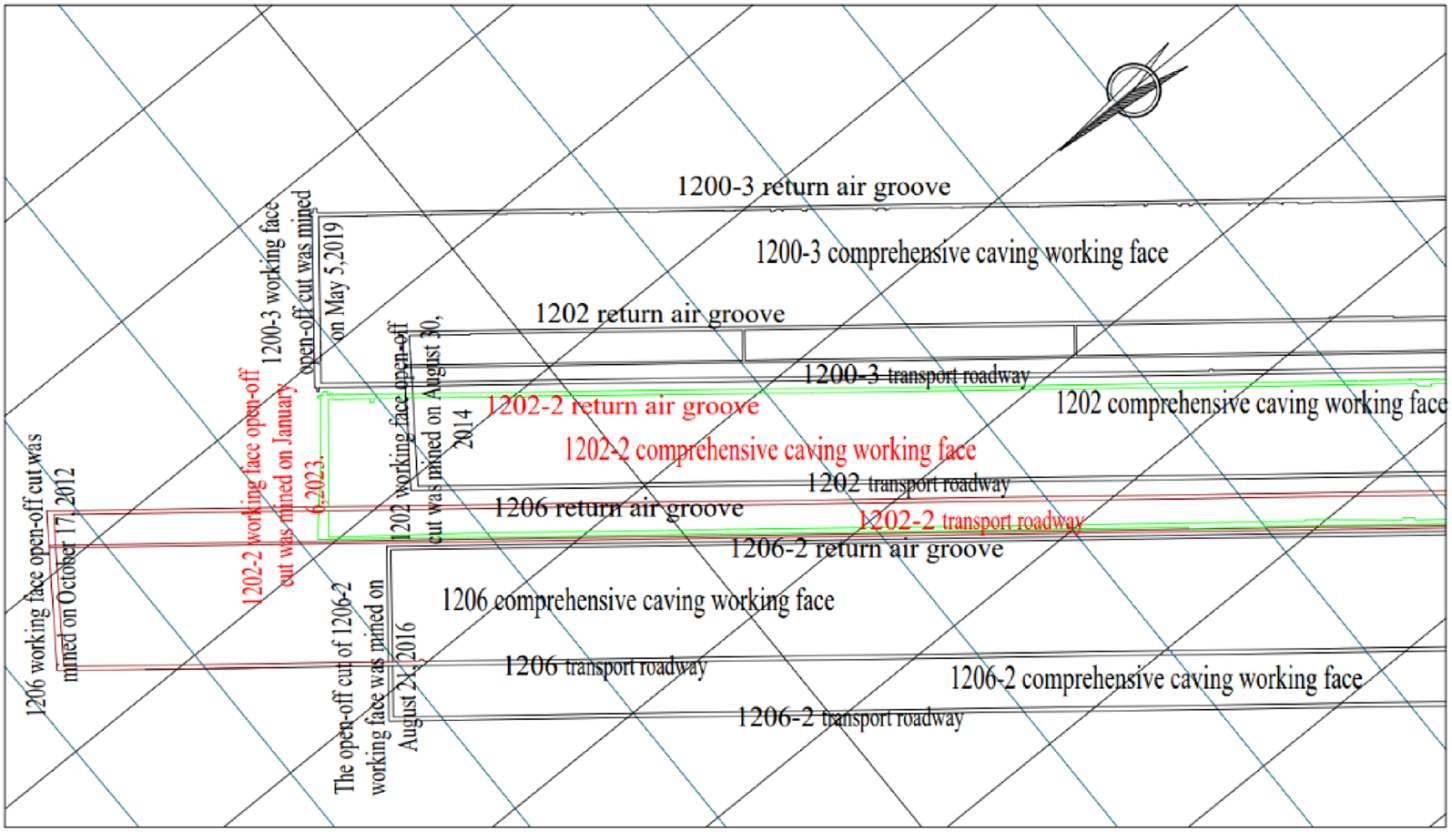
Baiyinhua mine mining engineering plan.

The 1202-2 workface is adjacent to the 1200-3 workface to the east, which will be completed in November 2022, and to the 1206 workface and 1206-2 workface to the west, which will be completed in September 2014 and June 2019, respectively, making the 1202-2 workface an isolated island workface. When the mining face advanced to 120 m, the protective layer was mined on the 1202-2 face, making the 1202-2 face a “C” island face, as shown in Fig. 1. The rock layer histogram is shown in Table 1.

**Table 1:** Rock strata histogram of 1202–2 working face.

Rock name	Thickness/m	Rock characteristics
Mudstone	58.5	Black mudstone mainly contains plant leaves and seed fossils
1st coal	40	Black brown coal, medium hardness coal, easy weathering, containing 1 m interlayer
Siltstone	3.3	The clastic components of siltstone are generally simple, mainly quartz, feldspar and debris are rare, and sometimes contain more muscovite
2 coal	1.6	The coal seam structure is relatively simple, mainly brown coal
Siltstone	5.3	Homogeneous in texture and containing plant leaf fossils
3 coal	2.1	It is dominated by dark brown coal, with dark coal bands
Mudstone	4.0	Mainly gray to dark gray, gray-black mud

## Characterization of coal seam determination and mineral pressure manifestation in very close-quarter mining

When the conditions of the coal seams in the mine and the spacing between the seams are different, the degree of influence on the mining of the coal seams is also different^15^. When the coal seam spacing is small to a certain extent, the lower coal seam working face roof is affected by the mining action of the upper coal seam working face, and the integrity of the lower coal seam roof is destroyed. Therefore, from the qualitative point of view of analysis, the biggest characteristic of very close distance mining is that the bearing layer has been affected by the mining action of the lower coal seam when the overall instability has occurred, which leads to the lower coal seam and the upper coal seam mining through the airspace, so whether the overall instability of the bearing layer occurs in the eye of the open cut is taken as the basis for the division of the very close distance mining of the coal seams.

When the upper coal seam is mined, the bottom plate stress changes from a stable state to an unstable state, and when the bottom plate stress is within the range of supporting pressure, shear damage is likely to occur, damaging the bottom plate^16^.

Before calculating the thickness of the bearing layer of the extremely close coal seam, we will first explain the symbols and definitions used, as shown in Table 2.

**Table 2:** Symbol definition table used in this paper.

Symbol	Definition
a	Open cut eye width
γ	Average capacity of overlying rock layers
σ	Penetration strength of base rock
*M*_c_	Average mining height
*K*_max_	Maximum stress concentration factor
*H*_m_	Average burial depth
*H*_L_	Critical bearing layer thickness
*H*_L_^′^	Critical effective bearing layer thickness
*H*_s_	Depth of damage to the bottom plate by mining stresses in the upper coal seam
*H*_x_	Thickness of the lower part of the bearing layer that naturally fell after the opening of the cutting eye

**Figure 2:**
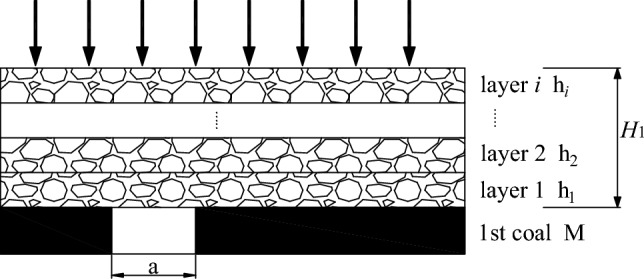
Mechanical model of coal seam roof in extremely close distance lower working face after upper coal seam mining.

As shown in Fig. 2, the thickness of the very close coal seam bearing layer is H_1_, the width of the open cut eye is a, the thickness of the lower coal seam is M, and h_1_, h_2_…h_*i*_ are the thickness of the interbedded layer in order.

Critical bearing layer thickness^17^$H_{L}$:

1 $$ H_{L} = H_{L} ^{\prime} + H_{s} + H_{x} $$

$H_{1}$ < $H_{L}$, it is a very close proximity mining.

2 $$ H_{L} = \frac{{\text{a}}}{2}\sqrt {\frac{{3\left[ {\gamma \left( {H_{{\text{m}}} - M_{{\text{c}}} } \right) + 59.88\ln \frac{{K_{\max } \gamma^{2} H_{{\text{m}}} }}{\sigma }} \right]}}{{R_{{\text{t}}} }}}  + 59.88\ln \frac{{K_{\max } \gamma H_{{\text{m}}} }}{\sigma } + H_{{\text{x}}} $$

${\text{a}}$ take 8.5 m, γ take 23 KN/m^3^, $H_{{\text{m}}}$ take 242 m, $M_{c}$ take 18 m, $K_{{{\text{max}}}}$ take 1.27, $\sigma$ take 6.9 MPa, $R_{{\text{t}}}$ take 0.3 MPa, $H_{x}$ there is no false roof in the roof of 1202-2 working face, take 0.

Calculated:

3 $$ H_{L} = 4.5 \times 0.013 + 1.45 \approx 1.51m > H_{1} $$

$H_{1}$ < $H_{L}$, the upper and lower coal seams are mined in extremely close proximity to each other. Therefore, the relatively stable “masonry beam” structure formed above the 1202-2 face in the process of mining back in the hollow area was broken and collapsed again, which led to the subsidence of the overlying fissure rock layer^18^. In the process of mining the lower coal seam, the supporting effect on the overlying rock layer is greatly weakened, and the resulting high stress is transferred to the working face and the overriding coal body, and the working face is frequently pressurized and the intensity becomes larger, so it is necessary to study the characteristics of the working face under the condition of mining airspace above the very close coal seam.

## Numerical simulation study of surrounding rock stresses in an island working face

### Model building

**Figure 3:**
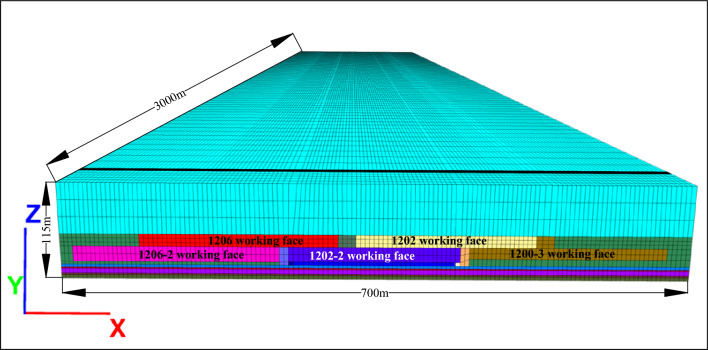
FLAC^3D^ simulation three-dimensional model of Baiyinhua coal mine.

Using FLAC^3D^ finite element analysis software, combined with Inner Mongolia Baiyinhua coal mine 1202-2 working face as the research background, to establish FLAC^3D^ synthesized island working face three-dimensional calculation model, in order to facilitate the comparison and calculation of convenience, will be slowly inclined to establish into the near-horizontal working face model, and the working face using the release of the top of the coal mining, the interbedded layer between the two working faces in the release of the top of the direct collapse of the formation of through the zone, so the Therefore, the interlayer between the two working faces is ignored when modeling to realize the simulation of top-loading mining, and the rest is exactly the same as the actual situation. The numerical simulation scheme is as follows: according to the project profile of 1202-2 working face, the Moore-Cullen model is established, and the length × width × height of the model is 3000 m × 700 m × 115 m. In order to make the simulation results and the real situation more closely match, the physical and mechanical parameters of coal rock are measured by using coal rock specimens collected from the site, and the physical and mechanical parameters of the coal rock are determined according to the engineering reality, as shown in Table 3. As shown in Table 3. As shown in Fig. 3. The boundary conditions are as follows: the left and right sides of the model are set with zero normal velocity, i.e., the displacement of the model boundary in the X-axis direction is zero; the front and rear sides of the model are set with zero normal velocity, i.e., the displacement of the model boundary in the Y-axis direction is zero; the bottom of the model is set with zero normal velocity, i.e., the displacement of the bottom of the model in the X-, Y-, and Z-axis directions is zero; the top boundary of the Z-axis is a free end, and the gravity of the model is set to be 9.80 m/s^2^ along the Z-axis in the negative direction.

**Table 3:** Physical and mechanical parameters of coal rock.

Rock sequence number	Rock name	Thickness *h*/m	Density *ρ*/KN·m^-3^	Unidirectional compressive strength *Rc*/MPa	Unidirectional tensile strength *Rt*/MPa	Elastic modulus *E*/GPa	Poisson ratio	Cohesion *C*/MPa	Internal friction angle *φ*/°
1	Mudstone	58.5	1760	12.49	0.28	13.7	0.29	0.15	34
2	1st coal	40	1216	10.24	0.26	2.2	0.24	0.16	30
3	Siltstone	3.3	22	8.45	0.21	9.5	0.27	0.14	42
4	2 coal	1.6	1324	9.74	0.24	2.2	0.23	0.21	32
5	Siltstone	5.3	2201	8.45	0.21	9.6	0.27	0.13	43
6	3 coal	2.1	1289	10.55	0.28	2.2	0.24	0.18	31
7	Mudstone	4.0	1863	11.28	0.21	13.7	0.28	0.15	34

The initial stress conditions were set as follows: a gradient stress of 8.57–9.53 MPa was applied along the X-axis direction; a gradient stress of 9.23–10.23 MPa was applied along the Y-axis direction; and a gradient stress of 6.59–7.33 MPa was applied at the top of the model along the Z-axis direction.

To make the simulation results closer to the actual situation, excavation is carried out according to the actual digging situation of the working face. First excavate 1206 working face, working face strike length 1970 m, inclined length 220 m; then excavate 1202 working face, working face strike length 2377 m, inclined length 200 m, 1202-2 working face is known as the left side and upper side of the neighboring empty island area; then excavate 1206-2 working face, working face strike length 2778 m, inclined length 220 m; finally excavate 1202-3 working face, working face strike length 2421 m, inclined length 220 m, resulting in the left and right sides of the face and the upper side of the island area. Then excavate 1206-2 working face, the working face has a strike length of 2778 m and an inclined length of 220 m. The left, right and upper sides of the mining face are adjacent to the empty area, which is in the shape of “C”, resulting in the working face being known as an island working face surrounded by high stress.

### Analysis of numerical simulation results

**Figure 4:**
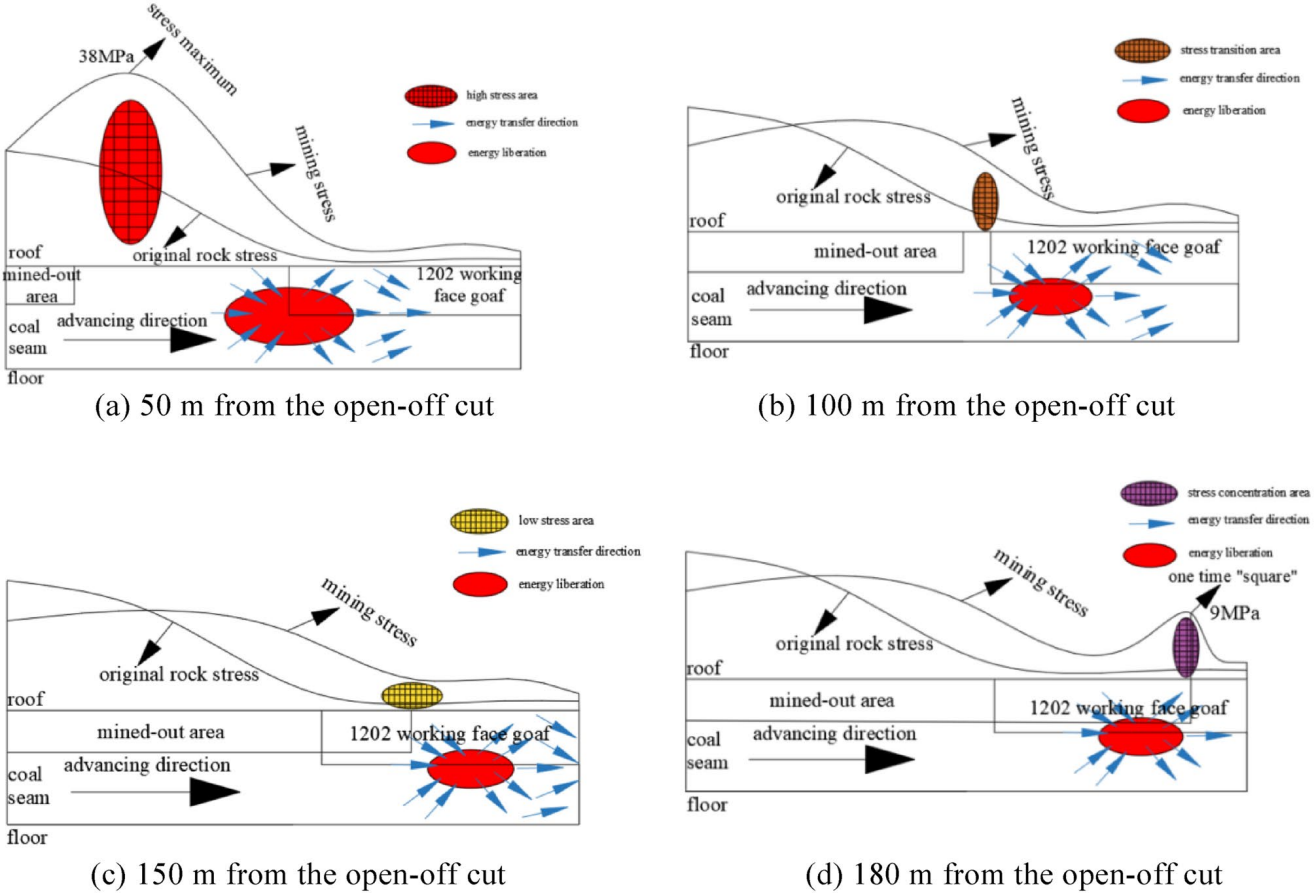
Stress cloud diagram of the working face at different mining positions.

As can be seen from Fig. 4a, when the island face is mined back to 50 m, a high-stress zone appears 5–10 m in front of the face, with a peak value of 38 MPa, and the roof stress at 50 m in front of the face enters into an unpressurized state. This is due to the mining of the overlying protective layer, the "red" energy release area in the figure, the release direction is consistent with the mining direction of the working face, resulting in the release of stress on the roof of the working face, and the roof of the working face enters the decompression state, and the mining stress is obviously reduced at this time.

As can be seen from Fig. 4b, when the isolated island working face is mined back to 100 m, the roof plate of the hollow area at the back of the working face bubbles down, which leads to the release of the accumulated energy of the high-stress area that appeared, and the final stress is not much different from the stress of the original rock, and the front of the working face is close to the upper protective layer of the mining area, which makes it become a stress transition area, and the stress is released.

From Fig. 4c, it can be seen that when the island face is mined back to 150 m after the upper protective layer is mined, the working face roof stress is released and stabilized, and this part of the area is called the low-stress zone.

As can be seen from Fig. 4d, when the island face was mined back to 180 m, the stress concentration in the face was 9 MPa due to the influence of the “square” and the mining movement factors, and the stress concentration was relieved after the “square”. After the “square”, the stress concentration phenomenon was relieved, and the stress gradually stabilized after decreasing, and the stress was at low stress.

## Comparative analysis of measured and simulated working resistance of hydraulic support

### Hydraulic support work resistance monitoring program

In order to be able to monitor the 1202-2 working face back mining process in different roof overburden state of mine pressure appearing law, in order to timely adjust the mining speed and take preventive measures. After installing the hydraulic support at the cutting eye, 10 YN-60 type mining seismic meters (0–80 MPa) were installed at the front lower column and rear upper column of hydraulic support No.1–100 to monitor the roof pressure in real-time.

### Comparative analysis of hydraulic bracket working resistance and simulation results

#### Analysis of working resistance law of hydraulic support under overlying coal rock body and comparison of simulation results

**Figure 5:**
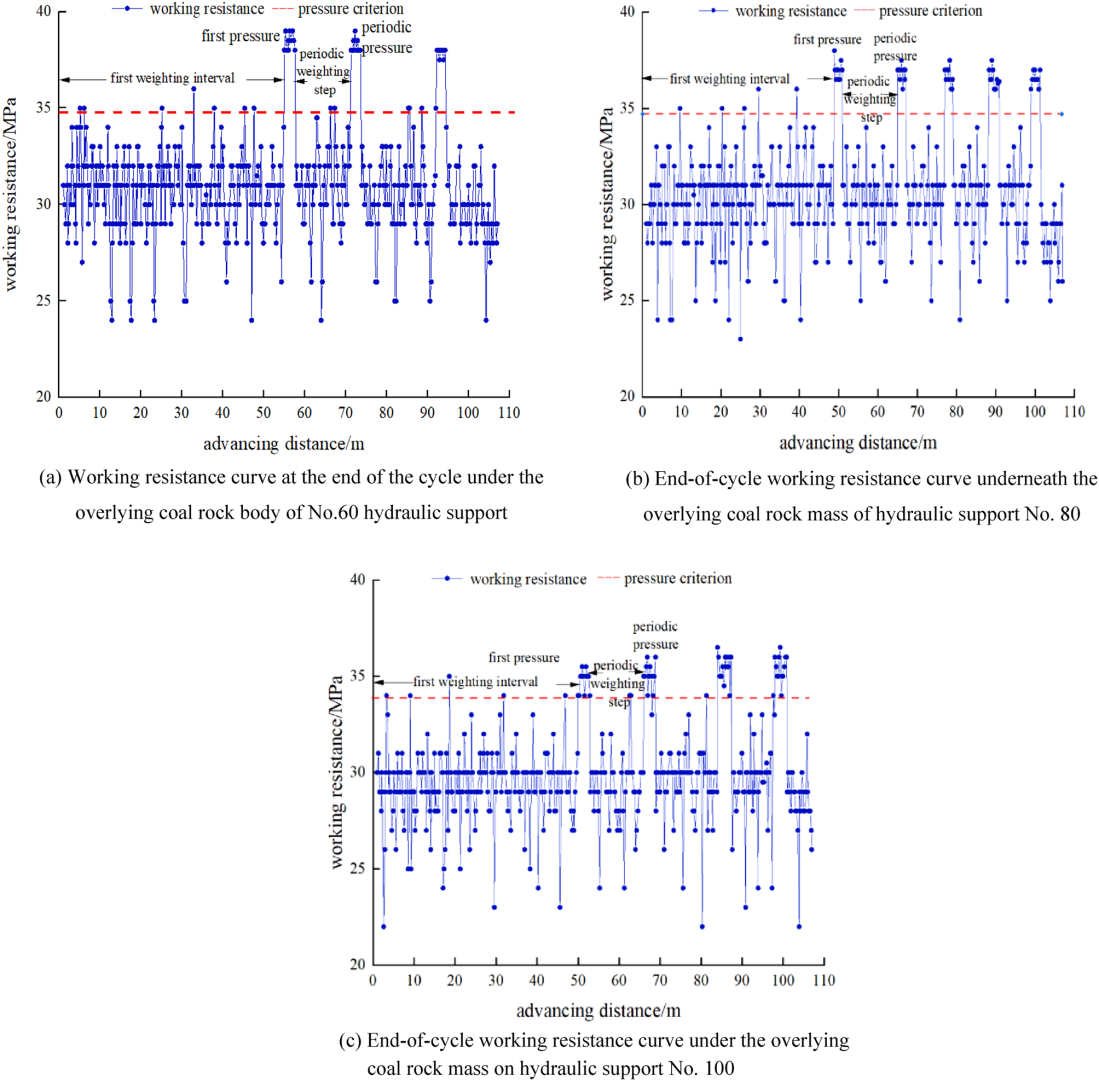
The working resistance curve of the hydraulic support at the end of the cycle in the working face under the overlying coal and rock mass.

With the continuous advancement of the mining face, the end-of-cycle working resistance of hydraulic braces No. 60, No. 80, and No. 100 under the overlying coal rock body is shown in Fig. 5.

With the continuous advancement of the mining face, the curve of the end-of-cycle working resistance of the hydraulic support under the overlying coal and rock mass is constantly fluctuating. The pressure strength of the goaf and the end-of-cycle working resistance of the hydraulic support are distributed in a ‘few’ shape in the tendency direction of the working face. The middle of the working face is the largest and gradually decreases to both ends, and the three have good consistency. There is no obvious law of the end-of-cycle working resistance of each hydraulic support in the strike length of the working face. The initial pressure step of No. 60 hydraulic support in the mining face is 55.7 m, the periodic pressure step is 20.0–23.3 m, with an average of 22.0 m, the average strength during the pressure period is 37 MPa, and the average working resistance of the hydraulic support at the end of the cycle during the non-pressure period is 32 MPa, and the dynamic load coefficient is 1.16. The initial pressure step distance of No.80 hydraulic support is 48.9 m, the periodic pressure step distance is 9–17.2 m, the average is 15.7 m, the average strength during the pressure period is 36 MPa, the average working resistance of the hydraulic support at the end of the cycle during the non-pressure period is 31 MPa, and the dynamic load coefficient is 1.16. The initial pressure step distance of No.100 hydraulic support is 50.5 m, the periodic pressure step distance is 9.3–20.4 m, the average is 16.0 m, the average strength during the pressure period is 33 MPa, the average working resistance of the hydraulic support at the end of the cycle during the non-pressure period is 29 MPa, and the dynamic load coefficient is 1.14.

The comparison between the FLAC^3D^ numerical simulation results and the measured working resistance of the hydraulic support shows that the stress peak deviation is 2.6%. With the continuous advancement of the working face, the upper part of the working face is close to the upper protective layer mining area, and the stress situation of the two is gradually relieved and has good consistency.

#### Analysis of working resistance law of hydraulic support under overlying goaf and comparison of simulation results

**Figure 6:**
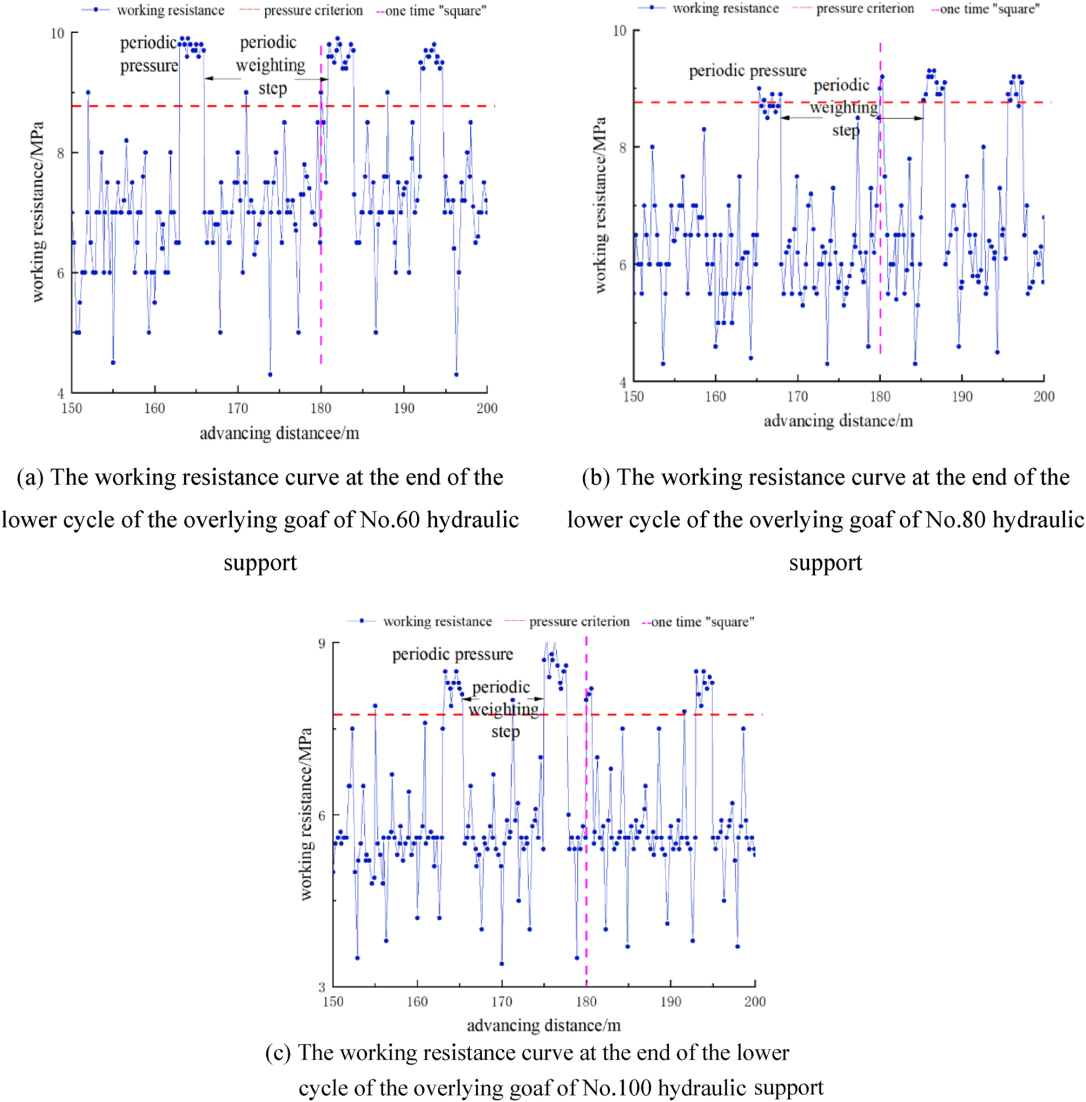
End-of-cycle working resistance curve of hydraulic support in working face under overlying goaf.

Figure 6 shows the working resistance curves of No.60, No.80, and No.100 hydraulic supports at the end of the cycle in the overlying goaf. Due to the mining of the upper protective layer, the working resistance of the support is greatly reduced, and its distribution law is similar to that of the overlying coal and rock mass. In the tendency direction, the pressure strength of the goaf and the working resistance of the hydraulic support at the end of the cycle is characterized by large middle and small ends.

With the continuous advancement of the mining face, the end-of-cycle working resistance curve of the hydraulic support under the overlying goaf is constantly fluctuating, but compared with the end-of-cycle working resistance of the hydraulic support under the overlying coal and rock mass, it has obvious regularity and periodicity. The periodic weighting step distance of No.60 hydraulic support in the working face is 11.3–22.4 m, with an average of 15.6 m, the average strength during the weighting period is 9.8 MPa, the average working resistance of the hydraulic support at the end of the cycle during the non-pressure period is 7.3 MPa, and the dynamic load coefficient is 1.34. The periodic weighting step distance of No.80 hydraulic support is 9 ~ 15.9 m, with an average of 13.4 m. The average strength during the weighting period is 8.6 MPa. The average working resistance of the hydraulic support at the end of the cycle during the non-pressure period is 6.4 MPa, and the dynamic load coefficient is 1.34. The periodic weighting step distance of No.100 hydraulic support is 10.5–17.6 m, with an average of 14.5 m. The average strength during the weighting period is 7.8 MPa. The average working resistance of the hydraulic support at the end of the cycle during the non-pressure period is 5.5 MPa, and the dynamic load coefficient is 1.42. When the working face is advanced to 180 m, the working face mining ‘square’ causes the pressure strength of the working face under the overlying goaf to be large and the pressure is frequent. After the ‘square’, the pressure step distance is relatively reduced, and the periodic pressure strength has no obvious change.

By comparing the numerical simulation results with the measured data of the hydraulic support when the working face advances 150–200 m, it can be seen that the stress conditions of the two are greatly reduced compared with 0–100 m. With the continuous advancement of the working face, the stress situation tends to be stable, and when the working face is in a ‘square’, there is no significant difference between the two stress peaks, indicating that the numerical simulation results are in good agreement with the measured results.

### Distribution law of working resistance of hydraulic support at the end of circulation in the working face

According to the analysis of the measured data of 10 YN-60 mine seismic tables installed, the mine pressure behavior in the mining process of the working face is shown in Table 4.

**Table 4:** Working resistance parameters of No.1–100 hydraulic support at the end of cycle.

Support number	Under the overlying coal rock mass				Under the overlying goaf		
	First weighting interval/m	Periodic weighting step/m	Ground pressure strength /MPa	Dynamic factor	Periodic weighting step/m	Ground pressure strength/MPa	Dynamic factor
1	43.7	16.3	31	1.11	15.6	6.8	1.43
10	46.3	15.7	33	1.13	13.7	7.3	1.21
20	49.5	20.1	32	1.12	21.6	6.9	1.32
30	48.2	16.5	32	1.18	16.8	7.2	1.26
40	49.6	18.1	34	1.14	17.6	7.6	1.33
50	54.8	20.8	36	1.16	16.2	8.3	1.29
60	55.7	22.0	37	1.16	15.6	9.8	1.34
70	53.6	19.8	34	1.14	16.8	9.8	1.26
80	48.9	15.7	36	1.16	13.4	8.6	1.34
90	49.5	17.6	31	1.13	16.3	7.5	1.33
100	50.5	16.0	33	1.14	14.5	7.8	1.42
Average value	50.03	18.05	33.55	1.14	16.19	7.96	1.32

Under the condition of mining under extremely close goaf, due to the poor caving state of the roof under the condition of fully mechanized caving, the different block structures caused by the caving caused different weighting steps, resulting in poor regularity of the first weighting and periodic weighting of the lower coal seam working face.

**Figure 7:**
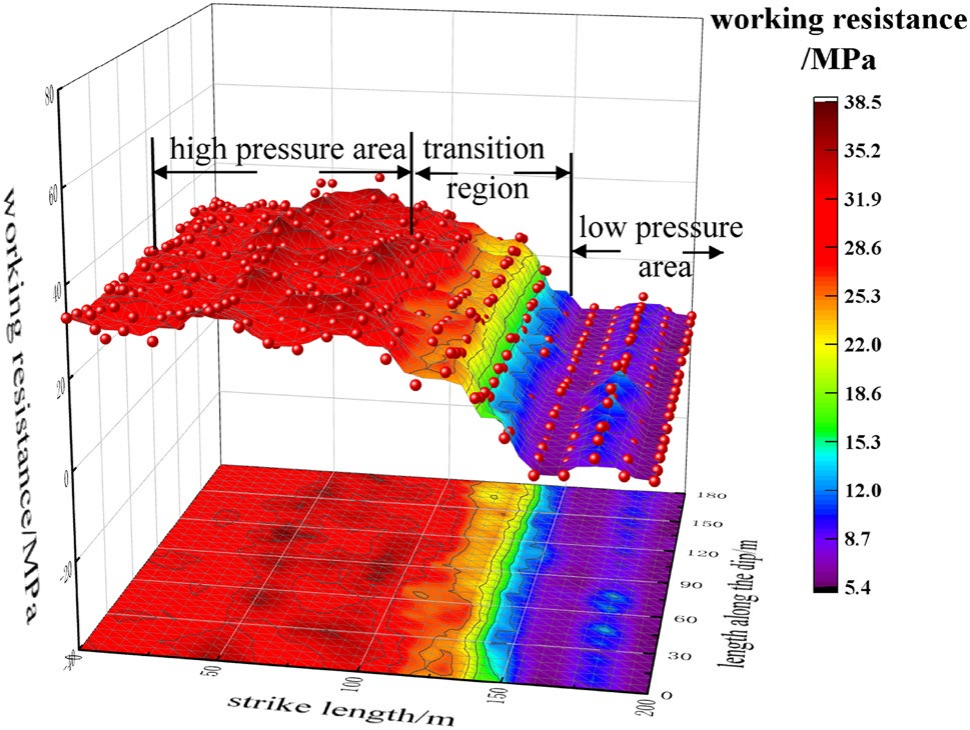
Stress distribution cloud diagram of working face.

As shown in Fig. 7, the working face is divided into three parts along the strike direction. (1) In the high-pressure area, when the working face advances 0–100 m, the upper protective layer is not mined and the working face is in the island working face state. During the non-pressure period, the working resistance of the hydraulic support at the end of the cycle does not fluctuate much, but it is basically about 32 MPa. (2) With the continuous advancement of the working face, the working face gradually becomes a three-sided adjacent working face. Due to the mining of the upper protective layer, the roof stress is gradually released, and the working resistance of the hydraulic support at the end of the cycle gradually decreases. In the low-pressure area, the working face advances 150 m backward to the low-pressure area. (3) After the working face ‘sees the square’, the pressure step distance is relatively reduced. During the non-pressure period, the working resistance of the hydraulic support tends to be stable at the end of the cycle, and there is no obvious floor heave and coal wall spalling in the working face.

## Conclusion

Through FLAC^3D^ numerical simulation, the mining stress and original rock stress curves of the working face to 50 m, 100 m, 150 m, and 180 m are analyzed respectively, and the mining stress is divided into four areas: high-stress area, stress transition area, low-stress area and ‘square’ stress concentration area.

In the field measurement, the first weighting step distance, periodic weighting step distance, weighting strength and dynamic load coefficient of the working resistance of No.60, No.80, and No.100 hydraulic supports at the end of the cycle under the overlying coal and rock mass and the overlying goaf are analyzed respectively. Under the overlying coal and rock mass, the first weighting step distance of the working face is 48.9–55.7 m, the peak pressure is 38 MPa, the periodic weighting step distance is 9.0–23.3 m, the peak pressure is 36 MPa, and the dynamic load coefficient of the working face is 1.14–1.16. Compared with the overlying coal and rock mass, the periodic weighting step under the overlying goaf decreases by nearly 10% on average, and the dynamic load coefficient increases by 20% on average. Compared with the measured results, the numerical simulation results are in good agreement with the measured results, which verifies that the numerical simulation results have a certain authenticity and rationality.

The working face can be divided into three parts along the strike direction: (1) the working face mining 0–100 m is a high-pressure area, the working face is under the overlying coal rock mass, the floor heave phenomenon is serious, and the coal wall spalling phenomenon is obvious. (2) When the working face is mined to 100–150 m, it is the transition zone. Due to the mining of the upper protective layer, the working face becomes a ‘C’ island working face, the roof stress is released, and the working resistance of the hydraulic support at the end of the cycle is greatly reduced. (3) After the working face is mined to 180 m, it is a low-pressure area. The working resistance of the hydraulic support tends to be stable at the end of the cycle, and there is no obvious floor heave and coal wall spalling in the working face.

The mining pressure of the isolated island working face under the extremely close goaf of the shallowly buried coal seam is strong. The monitoring and management of the roof pressure of the working face and the roadway should be strengthened to ensure the safe and efficient production of the working face.

## Data Availability

All data generated or analysed during this study are included in this published article.
